# The Power of First Steps

**DOI:** 10.3201/eid2508.AC2508

**Published:** 2019-08

**Authors:** Byron Breedlove, Kathleen Gensheimer

**Affiliations:** Centers for Disease Control and Prevention, Atlanta, Georgia, USA (B. Breedlove);; Food and Drug Administration, Silver Spring, Maryland, USA (K. Gensheimer)

**Keywords:** art science connection, emerging infectious diseases, art and medicine, about the cover, public health, listeriosis, Listeria monocytogenes, malaria, Zika, pregnancy, pregnant women, maternal health, food safety, viruses, bacteria, parasites, protozoa, Vincent van Gogh, first steps, after Millet, the power of first steps

**Figure:**
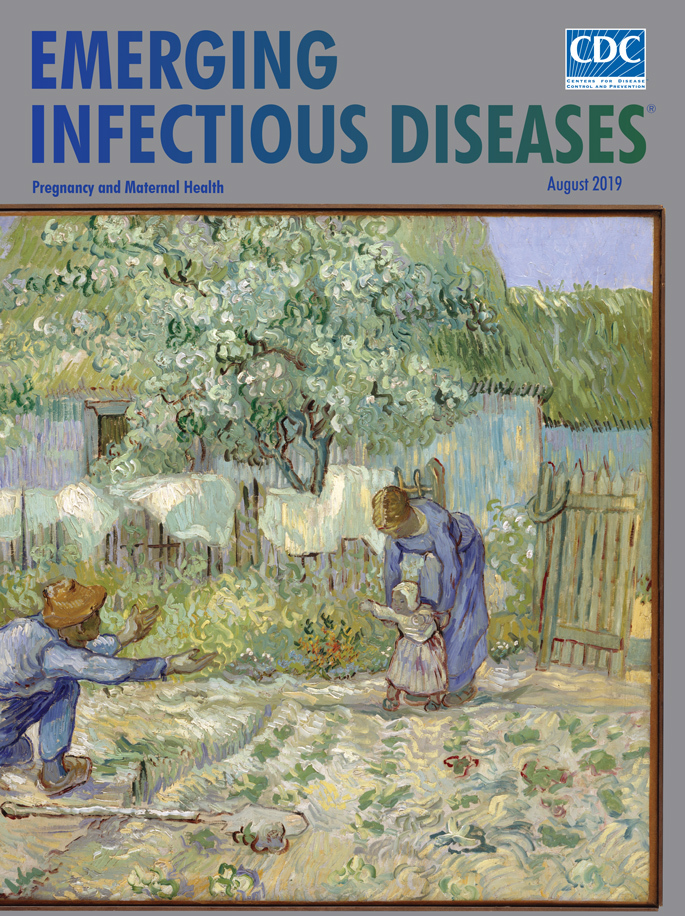
**Vincent van Gogh (1853–1890), First Steps, after Millet.** Oil on canvas; 28 1/2 in × 35 7/8 in/72.4 cm × 91.1 cm. Gift of George N. and Helen M. Richard, 1964. Image copyright © The Metropolitan Museum of Art. Image source: Art Resource, NY.

A number of paintings by the Dutch artist Vincent van Gogh feature children. One such painting, *First Steps, after Millet*, on this month’s cover, depicts a milestone moment for many parents and children. Father and daughter face each other with outstretched arms as the young child totters from her mother’s steadying hands. 

At the time van Gogh completed this work focusing on a child’s first steps, he was a voluntary resident of the Saint-Paul asylum in Saint-Rémy-de-Provence, France, where he lived and worked from May 1889 until June 1890. He sought care there after suffering a mental breakdown on December 23, 1888, and continuing to experience hallucinations and delusions. 

Despite several relapses of mental illness, van Gogh completed about 150 paintings during his time at the asylum. As noted by the Metropolitan Museum of Art, “His initial confinement to the grounds of the hospital is reflected in his imagery, from his depictions of its corridors to the irises and lilacs of its walled garden, visible from the window of the spare room he was allotted to use as a studio.” Later during his stay, he ventured outside the grounds, where he painted olive groves and cypresses. 

Van Gogh received black and white prints or photographs of works created by other artists from his younger brother Theo and used their content as his subjects. The Metropolitan Museum of Art explains that van Gogh considered his copies of other artists’ works to be “interpretations” or “translations,” and he compared “his role as an artist to that of a musician playing music written by another composer.” Working in his improvised studio in a barred cell, he would select a black-and-white image as his subject and "improvise color on it."

Prominent among those sources were works by the French painter Jean-François Millet, an artist who had influenced van Gogh’s decision to paint scenes from rural life. In all, van Gogh completed 21 paintings copied from works by Millet, including *First Steps, after Millet*. Millet’s *First Steps* is a pre-Impressionist black crayon sketch on tan paper. 

In the painting, van Gogh remained largely faithful to Millet’s drawing—the clothes drying on the fence; the spade laying across the furrows in the soil; and the posture and gestures of the man, woman, and child. He changed the perspective somewhat, revealing the sky, adding a gate, increasing the distance between the figures. In conveying the universal emotion of a tender moment, van Gogh does not focus on facial details. He painted his translation with characteristic brisk, hooked, curved strokes and relied on muted shades of browns, blues, and greens and wisps of white, in contrast to Millet’s lines that are more flowing and use only a monochromatic pallet. 

*First Steps, after Millet* reminds us of the stakes in protecting the health of mothers and children from infectious diseases, many of which are preventable and have receded because of public health efforts and medical breakthroughs. *Achievements in Public Health, 1900–1999: Healthier Mothers and Babies* reported that, at the beginning of the 20th century, in the United States—a decade after van Gogh completed his painting―for every 1,000 live births, approximately 100 infants died before 1 year of age and 6 to 9 women died of pregnancy-related complications. During 1915–1997, the infant mortality rate dropped more than 90%, from 100 to 7.2 per 1,000 live births; during 1900–1997, the maternal mortality rate declined almost 99%, to less than 0.1 reported deaths. 

Despite such progress, mothers and children continue to be at risk for emerging and reemerging infectious diseases globally and within the United States. Four diseases provide examples. In 2015–2016, the unanticipated and abrupt occurrence of Zika infections was linked to an increase in severe birth defects in affected regions. The full effect of the teratogenic potential for this vectorborne disease is still not fully understood. Malaria**,** another vectorborne disease**,** disproportionally affects infants and children under 5 years of age and pregnant women. Pregnant women who have malaria experience higher rates of miscarriage, intrauterine demise, premature delivery, low-birth-weight neonates, and neonatal death. Another example is Ebola virus infection. Although no evidence suggests that pregnant women are more susceptible to infection from Ebola virus than the general population, limited evidence does suggest that pregnant women are more likely to be at increased risk than the general population for severe illness and death when infected. *Listeria monocytogenes*, an important foodborne pathogen in the United States, provides the fourth example. Pregnant women, fetuses, and newborns are more likely than others to acquire invasive listeriosis, which can result in stillbirth, preterm labor, newborn sepsis, and meningitis. 

Other diseases also illustrate the continuing threat emerging and reemerging infectious diseases pose to mothers and children. Annual influenza seasons, including the 2009 H1N1 influenza pandemic, highlight the risks to pregnant and puerperal women, who may disproportionately become ill and die. Sometimes the rise in a noninfectious condition causes infectious diseases to reemerge and threaten maternal and child health. For instance, the US opioid epidemic has resulted in mothers becoming infected with hepatitis C, HIV, syphilis, and other sexually transmitted diseases. When those infections occur in mothers, they can also be transmitted to newborns. 

Death rates for mothers and children are generally higher in developing countries than in developed nations. Each step we take helps create a stronger global culture promoting the health of mothers and their young children. Even steps as small as those metaphorically depicted by van Gogh are vital for supporting public health efforts to promote maternal and child health.
